# Zearalenone Exposure Affects the Keap1–Nrf2 Signaling Pathway and Glucose Nutrient Absorption Related Genes of Porcine Jejunal Epithelial Cells

**DOI:** 10.3390/toxins14110793

**Published:** 2022-11-14

**Authors:** Qun Cheng, Shuzhen Jiang, Libo Huang, Yuxi Wang, Weiren Yang

**Affiliations:** 1 Department of Animal Sciences and Technology, Qingdao Agricultural University, Qingdao 266109, China; 202101040@qau.edu.cn; 2 Department of Animal Sciences and Technology, Shandong Agricultural University, Taian 271018, China; shuzhen305@163.com (S.J.); huanglibo227@126.com (L.H.); 3 Agriculture and Agri-Food Canada, Lethbridge Research and Development Centre, Lethbridge, AB T1J 4B1, Canada; yuxi_wang@hotmail.com

**Keywords:** zearalenone, intestinal porcine jejunal epithelial cells, Keap1-Nrf2 signaling pathway, oxidative stress, glucose nutrient absorption

## Abstract

This study aims to examine the impact of zearalenone (ZEA) on glucose nutrient absorption and the role of the Kelch-like erythroid cell-derived protein with CNC homology-associated protein 1 (Keap1)–nuclear factor erythroid 2-related factor 2 (Nrf2) signaling pathway in zearalenone-induced oxidative stress of porcine jejunal epithelial cells (IPEC-J2). For 24 and 36 h, the IPEC-J2 cells were exposed to ZEA at concentrations of 0, 10, 20, and 40 (Control, ZEA10, ZEA20, ZEA40) mol/L. With the increase of ZEA concentration and prolongation of the action time, the apoptosis rate and malondialdehyde level and relative expression of sodium-dependent glucose co-transporter 1 (Sglt1), glucose transporter 2 (Glut2), Nrf2, quinone oxidoreductase 1 (Nqo1), and hemeoxygenase 1 (Ho1) at mRNA and protein level, fluorescence intensity of Nrf2 and reactive oxygen species increased significantly (*p* < 0.05), total superoxide dismutase and glutathione peroxidase activities and relative expression of Keap1 at mRNA and protein level, fluorescence intensity of Sglt1 around the cytoplasm and the cell membrane of IPEC-J2 reduced significantly (*p* < 0.05). In conclusion, ZEA can impact glucose absorption by affecting the expression of Sglt1 and Glut2, and ZEA can activate the Keap1-Nrf2 signaling pathway by enhancing Nrf2, Nqo1, and Ho1 expression of IPEC-J2.

## 1. Introduction

Zearalenone (ZEA), a mycotoxin with an estrogen-like structure, competes with 17-estradiol for the estrogen receptor in target cells, impairing fertility, the ability to reproduce, and overall health [1]. α-zearalenol (α-ZEL), and β-zearalenol (β-ZEL) are metabolites of zearalenone that coexist in nature in grains and other foods. The harmful consequences of ZEA include carcinogenicity, nephrotoxicity, hepatotoxicity, endocrine disruptors, mutagenesis, and genotoxicity and are connected to alterations in endocrine disruptors and their metabolites (α-ZEL and β-ZEL) [2,3]. Since ZEA has an estrogen-like structure, studies have found that there are many estrogen receptor-positive cells in the intestine, so ZEA may also affect the function and integrity of the intestine [4,5]. Studies have demonstrated that when experimental animals are fed ZEA-contaminated feed, the intestine, which serves as the first line of defense against natural toxins, is exposed to ZEA and becomes the primary target organ for ZEA [6]. ZEA can alter intestinal villi structure [7,8], affect the integrity of porcine intestinal epithelial cells [9], and guide significant changes in gene expression levels of porcine intestinal cells [10]. We have found, in vivo, that ZEA can induce intestinal tissue damage, cause intestinal oxidative stress, and activate the Kelch-like erythroid cell-derived protein with CNC homology-associated protein 1 (Keap1)-nuclear factor erythroid 2-related factor 2 (Nrf2) signal pathway in post-weaning piglets [11,12,13]. Combining in vitro research is required to further demonstrate the potential contribution of the Keap1-Nrf2 signaling pathway to ZEA-induced intestinal oxidative stress since the internal environment governs the interaction and influence between various tissues and organs.

Glucose is the main energy substance for animal life activities and an intermediate product of animal body metabolism. Essential to its utilization is the intestine’s digestion and absorption [14,15]. The two primary transporters in the intestinal absorption of glucose are sodium-dependent glucose co-transporter 1 (Sglt1) and glucose transporter 2 (Glut2), making Sglt1 and Glut2 crucial for maintaining the homeostasis of the intestinal environment [16,17,18]. The intestinal epithelium absorption of nutrients may be influenced by the oxidative state across the gut lumen [19]. Studies have demonstrated that the degree of oxidative stress affects the stimulatory impact on Sglt1-mediated transport [19,20]. According to the research, mycotoxin lowered the levels of Sglt1 and Glut2 and caused abnormal expression of nutrient transporters in IPEC-J2 cells [21]. However, there are few reports about the barrier function of ZEA on cells and the absorption of glucose nutrients. The major goal of this study is to further investigate the oxidative stress toxicity of ZEA on cells and the effect on cell permeability and the glucose absorption capacity through in vivo experiments, thereby affecting animal health. At the same time, this study will provide new ideas and methods for the study of the toxicity of ZEA to nutrient absorption.

## 2. Results

### 2.1. IPEC-J2 Cells’ Morphology and Apoptosis

**Figure 1:**
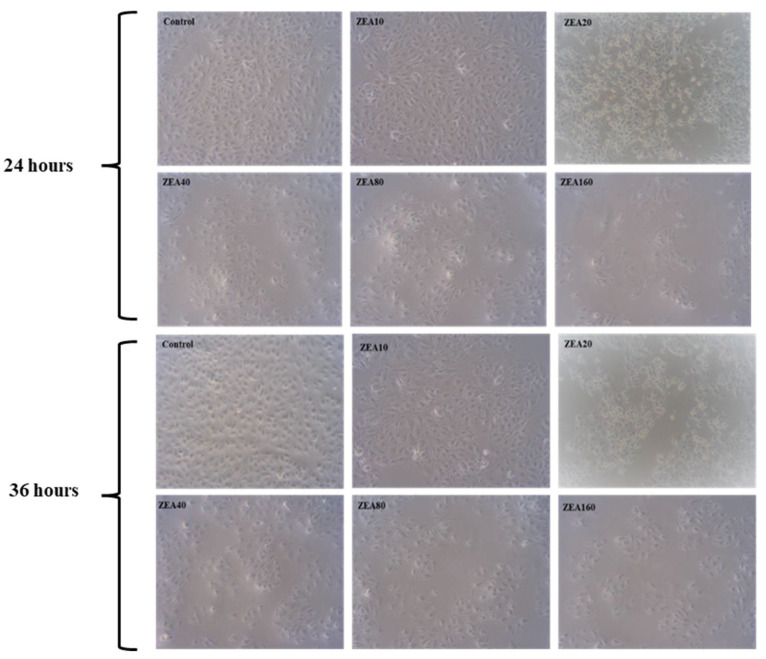
The morphology of intestinal porcine jejunal epithelial cells (IPEC-J2) exposed to ZEA at concentrations of 0 (Control), 10 (ZEA10), 20 (ZEA20), 40 (ZEA40), 80 (ZEA80) and 160 (ZEA160) μmol/L for 24 and 36 h and observed under a light microscope (10×).

As shown in Figure 1, the morphology of IPEC-J2 cells changed significantly with the extension of time and the increase of ZEA concentration. The results showed that after 24 h and 36 h, ZEA had no significant effect on the cells at low concentrations; when the concentration of ZEA reached 20 μmol/L, the cell viability decreased, and the cell morphology changed from normal paving stone to irregular shape; when the concentration of ZEA reached 80 and 160 μmol/L, the cells were destroyed seriously, and a large number of cells began to die and floated with extremely low activity.

**Figure 2:**
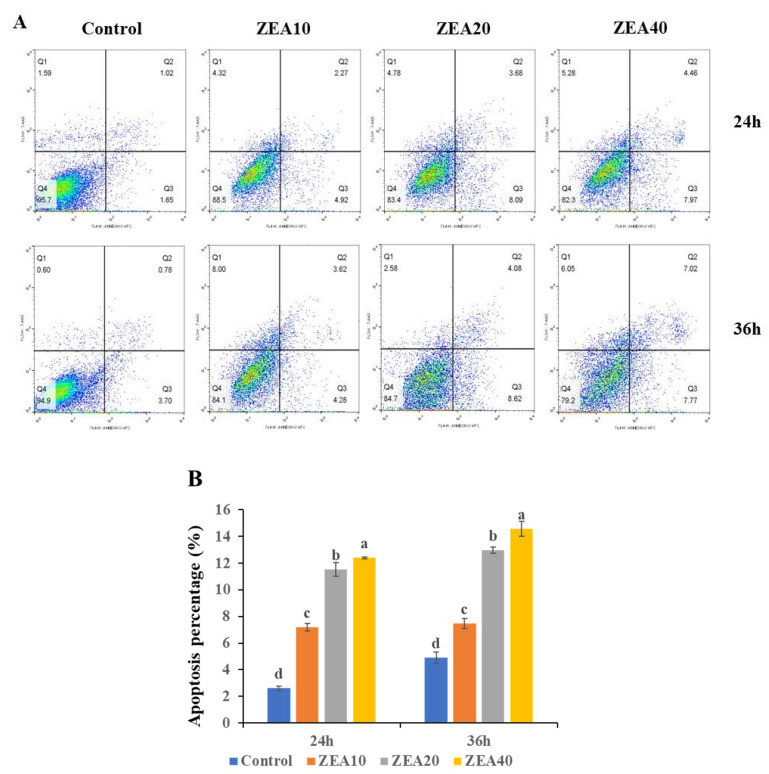
The apoptosis of intestinal porcine jejunal epithelial cells (IPEC-J2) exposed to ZEA at concentrations of 0, 10, 20, and 40 μmol/L (Control, ZEA10, ZEA20, ZEA40) for 24 and 36 h. (**A**) The apoptosis of IPEC-J2 cells was detected by flow cytometry. (**B**) The apoptosis percentage of IPEC-J2 cells. ^a–d^ The mean values differ significantly (*p* < 0.05).

With an increase in ZEA concentration and an extension of the action duration, IPEC-J2 cells’ apoptosis rate considerably increased after 24 and 36 h in comparison to the control group (Figure 2, *p* < 0.05). When the ZEA concentration reached 40 μmol/L and the cells were exposed for 36 h, the apoptosis rate reached the highest, indicating that the toxicity of ZEA on IPEC-J2 cells has a time and dose-dependent.

### 2.2. The TEER of IPEC-J2 Cells

**Figure 3:**
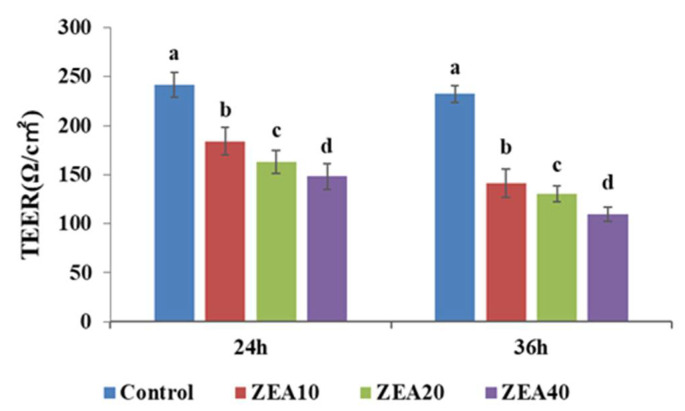
The transepithelial electrical resistance of intestinal porcine jejunal epithelial cells (IPEC-J2) exposed to ZEA at concentrations of 0, 10, 20, and 40 μmol/L (Control, ZEA10, ZEA20, ZEA40) for 24 and 36 h. ^a–d^ The mean values differ significantly (*p* < 0.05).

As shown in Figure 3, compared with the control group, the TEER value of IPEC-J2 cells was significantly reduced (*p* < 0.05) after culturing the cells with various concentrations of ZEA for 24 and 36 h. The TEER value of cells in the ZEA40 group is the lowest, and ZEA’s influence on TEER value of cells exhibits some time-dose dependence.

### 2.3. The Relative mRNA and Proteins Expression of Sglt1 and Glut2

**Figure 4:**
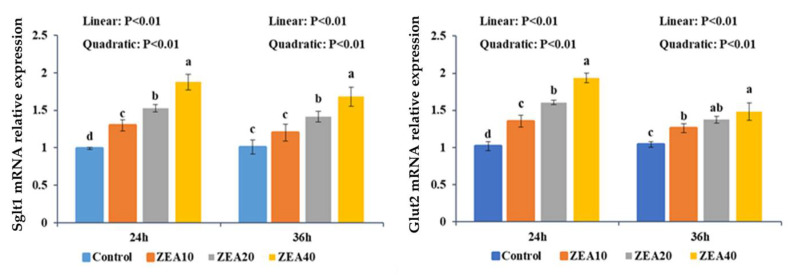
The relative mRNA expression of Sglt1, and Glut2 in intestinal porcine jejunal epithelial cells (IPEC-J2) exposed to ZEA at concentrations of 0, 10, 20, and 40 μmol/L (Control, ZEA10, ZEA20, ZEA40) for 24 and 36 h. ^a–d^ The mean values differ significantly (*p* < 0.05).

**Figure 5:**
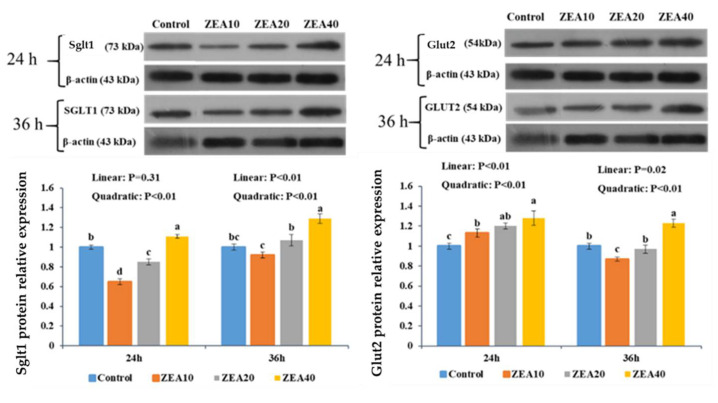
The relative protein expression of Sglt1, and Glut2 in intestinal porcine jejunal epithelial cells (IPEC-J2) exposed to ZEA at concentrations of 0, 10, 20, and 40 μmol/L (Control, ZEA10, ZEA20, ZEA40) for 24 and 36 h. ^a–d^ The mean values differ significantly (*p* < 0.05).

The relative expression levels of Sglt1 and Glut2 mRNA and protein in IPEC-J2 cells significantly increased linearly and quadratically with the rise in ZEA concentration at 24 and 36 h, respectively, as compared to the control group (Figure 4 and Figure 5, *p* < 0.05). The relative expression of Sglt1 protein in IPEC-J2 cells showed quadratically (*p* < 0.05) increase, with increasing ZEA concentration, and the expression level of Sglt1 protein increased significantly (*p* < 0.05) only in the ZEA40 group.

### 2.4. The Immunofluorescence Localization of Sglt1 in IPEC-J2 Cells

**Figure 6:**
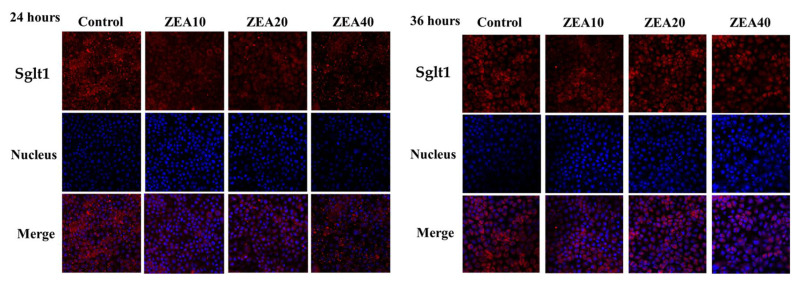
Immunostaining of Sglt1 on intestinal porcine jejunal epithelial cells (IPEC-J2) after exposure to to ZEA at concentrations of 0, 10, 20, and 40 μmol/L (Control, ZEA10, ZEA20, ZEA40) for 24 and 36 h, as detected under a light microscope (40×).

Immunofluorescence data obtained at 24 and 36 h showed that a large amount of Sglt1 is mostly dispersed in the cell membrane under normal conditions, and a minor amount is free in the cytoplasm (Figure 6). As the ZEA concentration rises, the fluorescence intensity around the nucleus decreases, indicating that the expression of Sglt1 in the cell membrane decreases, when the ZEA concentration reached 40 μmol/L, the expression of Sglt1 cell membrane was the lowest.

### 2.5. The Antioxidant Enzyme Activity of IPEC-J2 Cells

**Figure 7:**
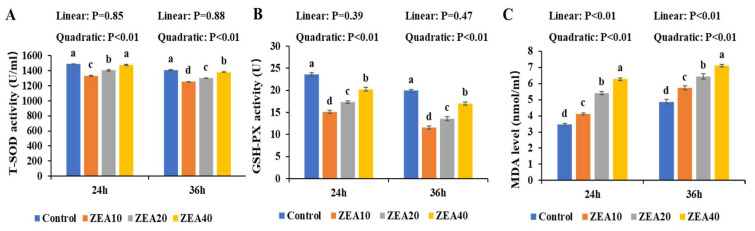
The antioxidant capacity of intestinal porcine jejunal epithelial cells (IPEC-J2) exposed to ZEA at concentrations of 0, 10, 20, and 40 μmol/L (Control, ZEA10, ZEA20, ZEA40) for 24 and 36 h. (**A**) The total superoxide dismutase (T-SOD) activities of IPEC-J2 cells. (**B**) The glu-tathione peroxidase (GSH-PX) activities of IPEC-J2 cells. (**C**) The malondialdehyde (MDA) content of IPEC-J2 cells. ^a–d^ The mean values differ significantly (*p* < 0.05).

In comparison to the control group, the T-SOD and GSH-PX activities of IPEC-J2 cells decreased quadratically with increasing ZEA concentration at 24 and 36 h (Figure 7, *p* < 0.05). IPEC-J2 cells exhibited the lowest T-SOD and GSH-PX activity at a ZEA concentration of 10 mol/L. At 24 h and 36 h, the MDA level of the cells in the ZEA treatment group increased linearly and quadratically with the increase of the ZEA concentration (*p* < 0.05). The MDA level was greatest in the ZEA40 group.

### 2.6. The Level ROS of IPEC-J2 Cells

**Figure 8:**
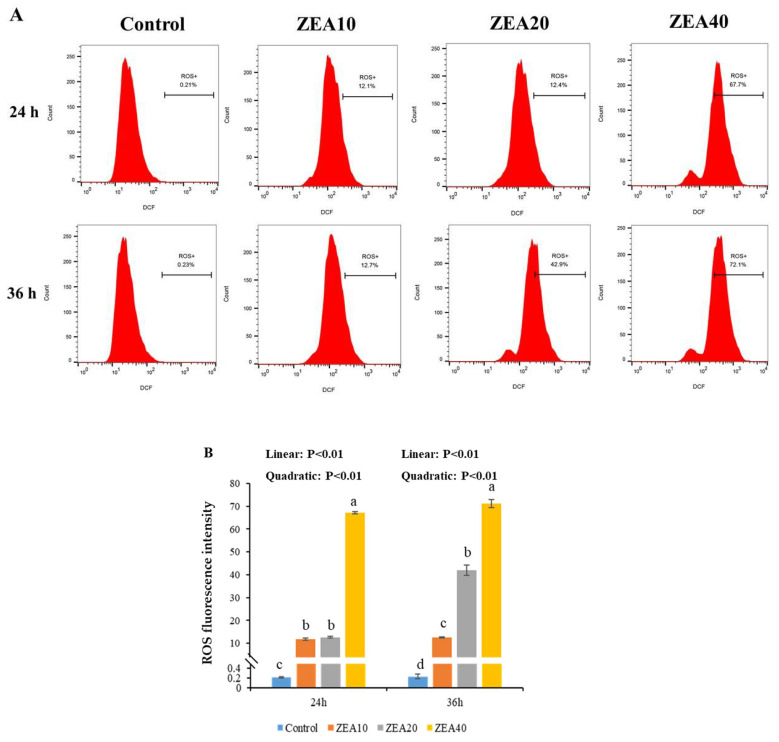
The reactive oxygen species of intestinal porcine jejunal epithelial cells (IPEC-J2) exposed to ZEA at concentrations of 0, 10, 20, and 40 μmol/L (Control, ZEA10, ZEA20, ZEA40) for 24 and 36 h. (**A**) The reactive oxygen species (ROS) of IPEC-J2 cells was detected by flow cytometry. (**B**) The ROS fluorescence intensity of IPEC-J2 cells. ^a–d^ The mean values differ significantly (*p* < 0.05).

Flow cytometer histogram analysis shows that at 24 h and 36 h, the peak value of DCF fluorescence intensity shifted to the right as ZEA concentration increased, and the mean fluorescence intensity (MFI) of ROS in the ZEA treatment group was significantly higher than in the control group (Figure 8, *p* < 0.05). At 36 h, the MFI value of ROS of IPEC-J2 cells in the ZEA40 treatment group was the highest, and the effect of ZEA on the MFI value of cellular ROS showed a certain time-dose dependence (Figure 8 A). At 24 h and 36 h, as the level of ZEA increased, the MFI value of ROS in IPEC-J2 cells increased linearly and quadratically (Figure 8B, *p* < 0.05).

### 2.7. The Relative Expression of Keap1, Nrf2, Nqo1, and Ho1 mRNA and Protein

**Figure 9:**
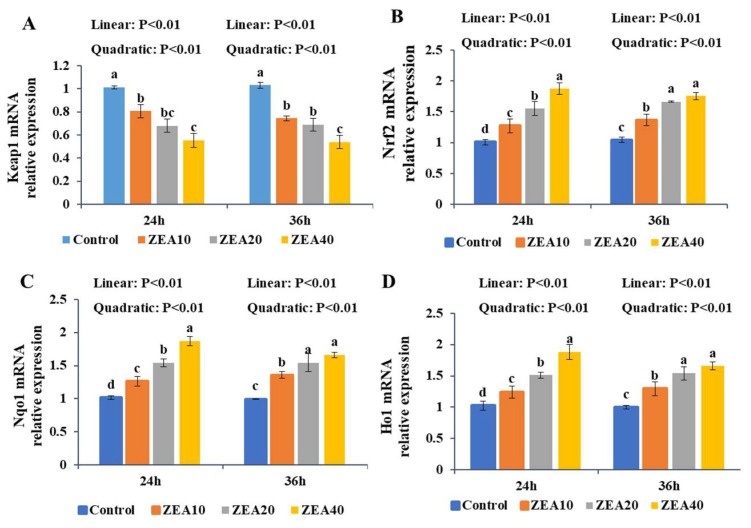
The relative mRNA expression of Keap1, Nrf2, Nqo1, and Ho1 in intestinal porcine jejunal epithelial cells (IPEC-J2) exposed to ZEA at concentrations of 0, 10, 20, and 40 μmol/L (Control, ZEA10, ZEA20, ZEA40) for 24 and 36 h. The Keap1 (**A**), Nrf2 (**B**), Nqo1 (**C**), and Ho1 (**D**) mRNA relative expression of IPEC-J2 cells. ^a–d^ The mean values differ significantly (*p* < 0.05).

**Figure 10:**
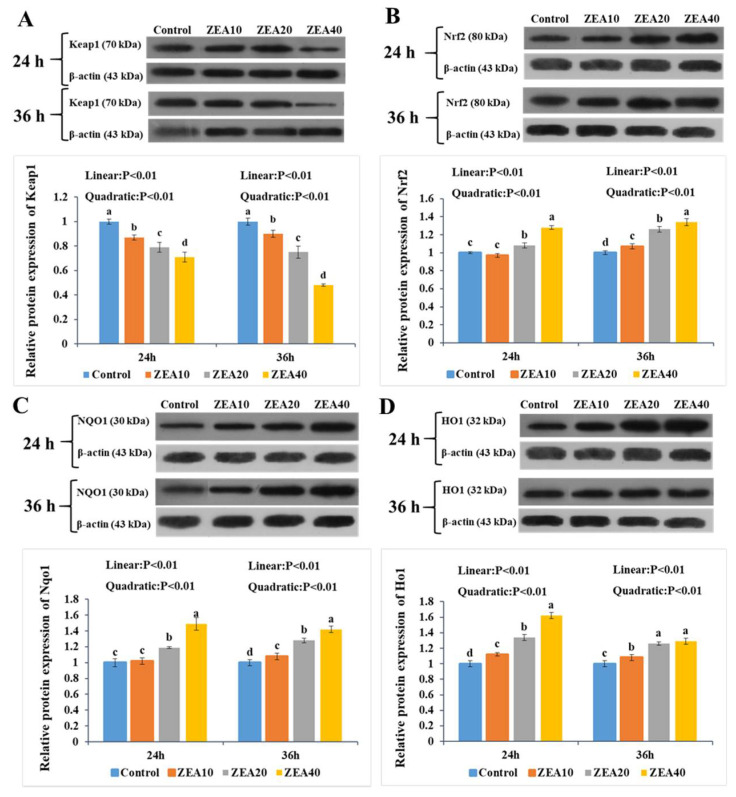
The relative protein expression of Keap1, Nrf2, Nqo1, and Ho1 in intestinal porcine jejunal epithelial cells (IPEC-J2) exposed to ZEA at concentrations of 0, 10, 20, and 40 μmol/L (Control, ZEA10, ZEA20, ZEA40) for 24 and 36 h. The Keap1 (**A**), Nrf2 (**B**), Nqo1 (**C**), and Ho1 (**D**) protein relative expression of IPEC-J2 cells. ^a–d^ The mean values differ significantly (*p* < 0.05).

At 24 h and 36 h, the relative expression of Keap1 mRNA and protein in IPEC-J2 cells treated with ZEA decreased linearly with increasing ZEA concentration relative to the control group (Figure 9 and Figure 10, *p* < 0.05). The ZEA40 group exhibited the lowest level of expression (Figure 9A and Figure 10A). The relative expression of Nrf2, Nqo1, and Ho1 mRNA and protein increased linearly and quadratically in IPEC-J2 cells at 24 and 36 h (*p* < 0.05). In the ZEA40 group, Nrf2, Nqo1, and Ho1 mRNA and protein expression levels are the highest (Figure 9B–D and Figure 10B–D).

### 2.8. The Immunofluorescence localization of ROS and Nrf2 in the IPEC-J2 Cells

**Figure 11:**
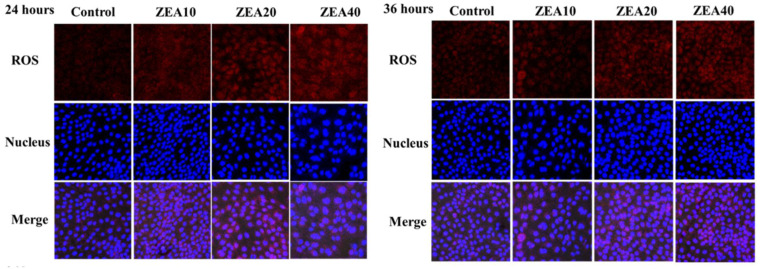
Immunostaining of ROS of intestinal porcine jejunal epithelial cells (IPEC-J2) after exposure to to ZEA at concentrations of 0, 10, 20, and 40 μmol/L (Control, ZEA10, ZEA20, ZEA40) for 24 and 36 h, as detected under a light microscope (40×).

Immunofluorescence results showed that ROS was weakly expressed in the control group IPEC-J2 cells at 24 and 36 h, and mainly distributed in the nucleus and cytoplasm (Figure 11). Fluorescence intensity surrounding the nucleus rose as ZEA concentration increased, indicating that ROS expression increased significantly. At 36 h, when the ZEA concentration reached 40 μmol/L, the cell ROS expression reached the highest.

**Figure 12:**
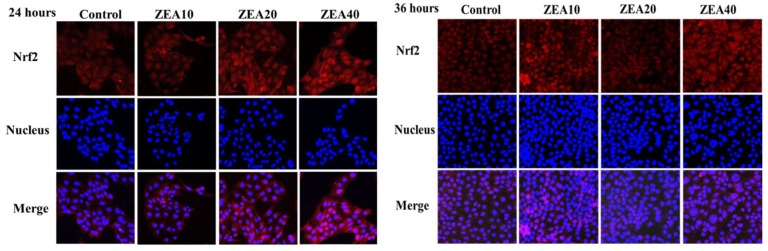
Immunostaining of Nrf2 of intestinal porcine jejunal epithelial cells (IPEC-J2) after exposure to to ZEA at concentrations of 0, 10, 20, and 40 μmol/L (Control, ZEA10, ZEA20, ZEA40) for 24 and 36 h, as detected under a light microscope (40×).

At 24 h and 36 h, the immunofluorescence results showed that under normal circumstances, Nrf2 was mostly localized in the cytoplasm and a tiny quantity in the nucleus (Figure 12). With the increase of ZEA concentration, the fluorescence intensity around and within the nucleus increased significantly, indicating that Nrf2 began to move to the nucleus, and the expression of Nrf2 increased significantly. At 36 h, when the ZEA concentration reaches 40 μmol/L, the expression of Nrf2 is the highest, and the phenomenon of Nrf2 invading the nucleus was very obvious.

## 3. Discussion

As we all know, zearalenone has toxic effects and can remain in the body for a long time after being absorbed by animals and humans, which brings huge losses to animal husbandry and poses a threat to human health. Our research confirmed that ZEA can produce oxidative stress in the intestines of weaned pigs, damage the structure and morphology of intestinal villi and affect the healthy development of the intestinal tract. Therefore, we guess that ZEA will have a certain effect on the nutrient absorption of pigs. In order to establish a new theoretical foundation for more effectively addressing the toxic effect of ZEA on pig intestines, the IPEC-J2 cell line was chosen as the cell model in this study to investigate the toxicity of ZEA.

Apoptosis is a basic biological phenomenon of cells, which is activated, expressed and controlled by a succession of genes in the body. When the regulation of apoptosis is unbalanced, excessive apoptosis and death of cells will cause serious damage to the body, which frequently results in the onset of disorders like autoimmune diseases [22,23]. In this study, we found that ZEA significantly reduces the cell viability of IPEC-J2 cells in a time- and dose-dependent manner, causing cell apoptosis and even cell death. In vitro investigations have demonstrated that ZEA and its metabolites can interfere with cell function and even induce apoptosis [24,25]. Some studies have also revealed that ZEA promotes apoptosis in rat germ cells, and after 12 h of ZEA treatment, the most apoptotic cells were detected, and then gradually decreased [26,27]. HepG2 cells were treated with ZEA at concentrations ranging from 7.1–250 M for 24 h, and the findings demonstrated a dose-dependent reduction in cell viability [28]. However, some studies have shown that ZEA (500 μg/L) cannot induce IPEC-J2 cell apoptosis [29], and ZEA (10–40 μM) cannot induce porcine kidney cell apoptosis [30]. This may be because various factors such as different cell types, exposure time, dose and type of mycotoxins, and metabolites affect cell viability [31,32].

The transmembrane resistance (TEER) of monolayer cells is one of the important indicators for studying the integrity of the intestinal tract, and its variations signify alterations in the permeability and integrity of the cell monolayer [33]. Some studies have shown that aflatoxin B1 (AFB1) and ochratoxin A (OTA) can reduce the TEER value of intestinal cells, resulting in increased permeability and a serious impact on the intestinal barrier function [34,35]. Deoxynivalenol (DON) has been shown to have impacts on the TEER of three distinct human intestinal epithelial cell lines, including HT-29, Caco-2, and T48, and it was shown that these effects were dose-dependent [33]. The similar result was also reached by DON’s investigation on the TEER value of the swine intestinal epithelial cell lines IPEC-1 and IPEC-J2 [36]. These studies’ findings and those of this one are comparable. This study found that ZEA significantly decreased the TEER of IPEC-J2 cells, and that this reduction in TEER was dose- and time-dependent. We analyzed that the decrease of TEER induced by ZEA may be due to the destruction of tight junction, the change of ion transmembrane transport and the oxidative stress induced by mycotoxin, and it may also be due to the oxidative stress induced by ZEA, which needs further research and verification.

The small intestine can be actively transported through the Sglt1 of the small intestinal mucosal epithelial cells to transport the glucose in the intestinal lumen of the small intestine to the epithelial cells, followed by Glut2 facilitating diffusion, which transfers the intracellular glucose to the blood [37]. According to studies, three distinct transporters involved in the intestinal absorption of glucose and fructose have varying susceptibility to oxidative stress. Sglt1 is the most sensitive, followed by Glut2, and Glut5 is the lowest [20]. Notably, an increase in oxidative stress can stimulate and promote Sglt1 and Glut2. Nonetheless, oxidative stress or situations associated to oxidative stress have been demonstrated to have detrimental effects on the Glut transporter [20,38,39]. This study revealed that the protein expression of Sglt1 and Glut2 was significantly reduced in low-dose ZEA group and increased significantly in high-dose ZEA group. Cellular immunofluorescence technology has been widely used in the fields of medicine and biology. It is a common method to detect the intracellular localization of antigen proteins and quantify their expression by using fluorescein-labeled antibodies by using the specific binding reaction between antigens and antibodies [40]. At the same time, the immunofluorescence results showed that Sglt1 expression in the cell membrane was considerably downregulated in ZEA treatment group. It is found that ZEA (10 μg/mL) induces oxidative damage in intestinal cells, impairs nutritional digestion and absorption, and increases the mRNA expression level of Sglt1 [41]. Studies have also found that the combination of 500 g/L ZEA and 40 g/L AFB1 may increase the expression of the Glut2 gene in IPEC-J2 cells [29]. Therefore, we think that different kinds and concentrations of toxins have different effects on the expression of Sglt1 and Glut2 in different cells and may have a certain relationship with the exposure time of toxins. In this study, we found that the up-regulation of Sglt1 and Glut2 mRNA expression levels was inconsistent with protein expression, indicating that low concentrations of ZEA increased the expression of Sglt1 and Glut2 mRNA, but did not promote the simultaneous increase in the transport activity of Sglt1 and Glut2. Some studies have explained this phenomenon. The activity of GLUTS is inhibited by one or more regulatory proteins with very short half-life. When protein synthesis is inhibited, the expression level of regulatory proteins decreases, and the inhibitory effect on GLUTS activity is lifted, resulting in the increase of mRNA expression and activity [41].

Oxidative stress in the organism can be brought on by an imbalance between antioxidant defense and ROS free radical generation [42]. ZEA is believed to be a potent inducer of ROS in the mammalian body, which can induce the massive production of ROS [43,44]. According to some reports, ZEA can induce ROS production and lipid peroxidation, and reduce cell proliferation. The oxidative stress that ZEA causes may be responsible for these cytotoxic effects [9,45,46]. In order to inhibit the increase of ROS and improve the ability of cells to resist oxidative stress damage, cells will activate their own antioxidant defense mechanisms, containing SOD and GSH-PX antioxidant enzymes [42,47,48]. Numerous in vivo and in vitro investigations have demonstrated that ZEA can reduce the activities of the antioxidant enzymes T-SOD and GSH-PX and increase the level of MDA by regulating the antioxidant mechanism in the cell [9,46,49]. The relative expression of ROS mRNA and protein as well as the MDA content in the cells were dramatically raised following ZEA treatment of IPEC-J2 cells for 24 h and 36 h in this investigation, whereas the activities of the antioxidant enzymes T-SOD and GSH-PX were significantly decreased. ZEA promotes a large number of cells in a state of oxidative stress, which is consistent with our previous research results in the pig intestine [11,12,13]. Some studies also found that the level of ROS in human embryonic kidney cells exposed to ZEA for 1 h did not change significantly, but it increased significantly after 2 h [50]. ZEA can promote cytotoxicity in rat intestinal epithelial cells by decreasing T-SOD and GSH-Px activities and elevating MDA levels [6]. Therefore, previous studies and our own have demonstrated that ROS induced by ZEA injury produces oxidative stress and reduces cellular antioxidant capacity, which may be an important reason for ZEA damage to cell homeostasis and structure, leading to cell death.

Under oxidative stress, the Keap1-Nrf2 signaling pathway is activated, and the conformational change of Keap1 in the cytoplasm shifts the Nrf2 released from the low binding site to the nucleus, triggering the binding of the programmed antioxidant enzyme and the phase II detoxifying enzyme to the ARE. This will produce proteins that protect cells, including antioxidant enzymes and Ho1, Nqo1, etc., so as to improve the body’s antioxidant capacity and playing an important role in maintaining cell homeostasis when cells are under oxidative stress [51,52]. The findings of this study showed that ZEA significantly increased the relative expression of Nrf2, Ho1 and Nqo1 mRNA and protein in IPEC-J2 cells, and significantly reduced the relative expression of Keap1 mRNA and protein. Meanwhile, Nrf2 immunofluorescence showed that ZEA significantly induced the increase of Nrf2 gene expression in the cells, and Nrf2 transferred from the cytoplasm to the nucleus. Nrf2 expression reached its maximum level in the ZEA40 treatment group, which was consistent with our previous results in the pig intestine [11,12,13]. As a result, we think that ZEA can activate the Keap1-Nrf2 signaling pathway of IPEC-J2 cells, and the activation of this pathway increases the ability of IPEC-J2 cells to resist ZEA toxicity. Studies have found that ZEA toxicity can significantly induce differences in gene expression in IPEC-1 cells, and low doses of ZEA (10 μmol/L) significantly upregulate the expression of 70% of genes in IPEC-1cells, including encoding Gpx [9,10]. Interestingly, despite the fact that the expression of Nrf2, Ho1, and Nqo1 genes increases significantly when this route is active, the response is restricted since ZEA-induced ROS also activates a variety of signal pathways such as cell death [53,54]. However, the influence of the Keap1-Nrf2 signaling pathway on the oxidative stress induced by ZEA in IPEC-J2 cells is poorly studied. In order to determine whether the increased expression of Ho1 and Nqo1 genes in IPEC-J2 cells caused by ZEA is controlled by the classic Keap1-Nrf2 signaling pathway alone or by various pathways, we will further use ShRNA to interfere with the expression of Nrf2 in cells to inhibit the activation of the Keap1-Nrf2 signaling pathway for verification experiments.

## 4. Conclusions

In conclusion, ZEA (10, 20, 40 μmol/L) changes the morphology of IPEC-J2 cells in a certain time- and dose-dependent manner, which changes the permeability of cells, interferes with the expression of Sglt1 and Glut2 genes, and may affect the glucose and nutrient absorption capacity of the cells. ZEA induces oxidative stress in IPEC-J2 cells, which reduces cell antioxidant capacity, and the Keap1-Nrf2 signaling pathway is activated to resist the toxic effects of ZEA during ZEA-induced cellular oxidative stress. However, the link between ZEA’s activation of the Keap1-Nrf2 signaling pathway and the mechanism by which ZEA modulates the expression of the Sglt1 and Glut2 genes influences cellular glucose food absorption requires additional investigation.

## 5. Materials and Methods

### 5.1. Preparation of IPEC-J2 Cells Culture

The IPEC-J2 cells were obtained commercially (Beina biological Co., Ltd., Beijing, China) The IPEC-J2 cells were cultured with 2 mL of Dulbecco’s Modified Eagle Medium (DMEM) high glucose (11995-065, GIBCO Co., Ltd., Shanghai, China) containing 10% fetal bovine serum (FBS, 16000-044, GIBCO Co., Ltd., Shanghai, China) and 1% Penicillin-Streptomycin (P/S, 15140-163, GIBCO Co., Ltd., Shanghai, China). Before being employed in each test, the incubation was carried out at 37 °C in a cell incubator with 5% CO_2_.

### 5.2. Preparation of ZEA Treatment of IPEC-J2 Cells

Commercially available zearalenone (Sigma, Z2125, MO, USA) was dissolved in dimethyl sulfoxide (DMSO) (Sigma, D2650, MO, USA) at a concentration of 20 mmol/L and refrigerated at −20 °C prior to use. The IPEC-J2 cells were seeded onto 6-well plates at a density of 1 × 10^6^ cells per well before aliquots of ZEA solution were added to achieve culture concentrations of 0, 10, 20, and 40 μmol/L (Control, ZEA10, ZEA20, ZEA40), respectively. After mixing the cultures, they were incubated at 37 °C for 24 and 36 h, after which the cells were collected for further testing.

### 5.3. Determination of Apoptosis in IPEC-J2 Cells

The cell samples were obtained, twice-washed with 2-mL of cold PBS, and re-suspended into 100 μL 1×Binding Buffer. The collected IPEC-J2 cells were then stained for 15 min at 25 °C in the dark with PI (Beyotime, C1052-2, Shanghai, China) and FITC Annexin V (Beyotime, C1052-3, Shanghai, China). After the reaction, add 400 μL of 1 × Binding Buffer to each tube and mix well, and then examined using a BD FACSCaliburTM flow cytometer (FACSCalibur, BD, San Jose, CA, USA) with Flowjo 7.6 software. 

### 5.4. Determination of Cell Transepithelial Electrical Resistance (TEER) of IPEC-J2 Cells

The cells collected and diluted to 5 × 10^3^ μL^−1^. Then 100 μL cell suspension was inoculated in the chamber of 12-hole Transwell plate (membrane area 1.12 cm^2^), then 400 μL complete medium was added, and 1.5 mL complete medium was added in the hole under the chamber. After placing the Transwell plate in the incubator for one day, a compact monolayer cell was formed, and then the complete medium was replaced with ZEA different treatment groups of medium, each treatment was repeated for three times. After 24 h and 36 h of treatment, the cells in each group were measured by cell resistance meter (Millipore Mers00002 Millicell ERS, MA, USA). Three points in different directions were selected for each hole.

### 5.5. Determination of Antioxidant Enzyme Activity

T-SOD A001-1, GSH-PX A005 and MDA A003 detection kits (Nanjing Aoqing Biotech-nology Co., Ltd., Nanjing, China) were used to assess the cell samples for malondialdehyde (MDA) content and total superoxide dismutase (T-SOD) and glu-tathione peroxidase (GSH-PX) activities [55].

### 5.6. Determinations of Relative mRNA Expressions

The total RNA from the cells was extracted using RNAiso Plus (Applied TaKaRa, Dalian, China) per the manufacturer’s instructions. Using an Eppendorf Bio-photometer (DS-11, Denovix, Wilmington, DE, USA) with an absorbance ratio of 260/208 nm, the purity and concentration of the RNA were assessed. A reverse transcription system kit (PrimeScriptTM RT Master Mix, RR036A, Applied TaKaRa, Dalian, China) was used to convert total RNA to cDNA, and the resulting cDNA was split into two subsamples.

The cDNA subsample was utilized for quantitative real-time PCR analysis. The qRT-PCR study employed a total volume of 20 µL of the PCR reaction mixture made up of 10 µL SYBRY Premix Ex Taq II, 0.4 µL DyeII (SYBRY Premix Ex Taq-TIi RNaseH Plus, DRR420A; Applied TaKaRa), 0.4 µL of both forward and reverse primers, and 2 µL cDNA (<100 ng). Sangon Biological Engineering Technology and Services Co. Ltd. (Shanghai, China) designed all of the primers, and the Beijing Genomics Institute synthesized them (BGI, Beijing, China). An initial denaturation phase at 95 °C for 30 s was followed by 43 cycles at 95 °C for 5 s, 60 °C for 34 s, 95 °C for 15 s, 60 °C for 60 s, and 95 °C for 15 s in the optimized qRT-PCR procedure. An AB 7500 Real Time PCR System was used to carry out the qRT-PCR experiments (Applied Biosystems, Foster City, CA, USA). Using the 2^−ΔΔCt^ method, the relative expression level of Sglt1, Glut2, Keap1, Nrf2, Nqo1, Ho1, and β-actin mRNA was determined [56]. For each sample, the analysis was done three times. Table 1 presents the primer sequences and production lengths.

### 5.7. Determination of Sglt1, ROS, and Nrf2 Distribution in IPEC-J2 Cells

The IPEC-J2 were initially seeded on microslides, which were then treated with ZEA as stated above, fixed with 4% paraformaldehyde for 1 h, then penetrated with 0.5% Triton X-100 for 10 min at room temperature. The resulting cells underwent the following processing steps: washing with PBS three times for 5 min each, blocking with 10% FBS for 1 h, Nrf2 (1:500, ab89443, Abcam, Cambridge, UK), reactive oxygen species (ROS, 1:200, ab236409, Abcam, Cambridge, UK), Sglt1 (1:150, ab247121, Abcam, Cambridge, UK) incubation at 4 °C overnight, three washings with PBS, mixing with goat anti-rabbit lgG that has been Alexa Fluor 555-labeled (1:200, ab150079, Abcam, Cambridge, UK) at 37 °C in the dark for 1 h, and washing with PBS. The appropriate Hoechst 33342 (C1022, Beyotime, Shanghai, China) was then added to the previously prepared cells, stirred for 5 min, and then rinsed with PBS. Under a confocal microscope, the treated samples were analyzed (FLUOVIEW FV3000, Olympus, Japan).

### 5.8. Determination of Protein Expression

The cell samples were then washed with 2 mL of PBS and centrifuged once more (1200× *g*, 5 min). Following the manufacturer’s instructions, they were extracted with lysate containing PMSF (1 mmol/L, Beyotime, Shanghai, China). Using a BCA protein assay kit (Beyotime, Shanghai, China), the total protein content of the extract was assessed before it was subjected to western blotting to detect the protein expression of relevant mRNA, as indicated below. Each sample was loaded with 60 μg of protein and put through 1.5 h of electrophoresis on polyacrylamide gels. After then, the bands that had been separated were moved to immobilon-p transfer membranes (So-larbio, Beijing, China). These membranes were first blocked in 10% skim milk powder for 2 h, then washed three times with Tris Buffered Saline Tween (TBST, pH 7.6), and finally incubated overnight at 4 °C with monoclonal mouse antibody β-actin (1:1500, SC-47778, Santa Cruz, CA, USA), polyclonal rabbit antibody Nrf2 (1:1000, ab92946, Abcam, Cambridge, UK), polyclonal rabbit antibody Keap1 (1:1000, ab196346, Abcam, UK), Nqo1 (1:500, ab2346, Abcam, UK), Ho1 (1:500, ab13248, Abcam, Cambridge, UK), Sglt1 (1:1000, bs-1128R, BIOSS, Beijing, China), Glut2 (1:500, bs-0351R, BIOSS, Beijing, China). Following the primary incubation, the membrane was washed with TBST three times for a total of 5 min each time, and then it was subjected to a secondary incubation with goat anti-rabbit lgG (1:5000, Thermo Pierce 31210, Thermo Fisher Scientific, MA, USA) and goat anti-mouse lgG (1:5000, Thermo Pierce 31160, Thermo Fisher Scientific, MA, USA) in diluted secondary antibody dilution buffer (Beyotime, Shanghai, China) at room temperature for 2 h. After 30 min of washing with TBST, the membranes were submerged in a high-sensitivity luminescence reagent (BeyoECL Plus; Beyotime Biotechnology). After that, the membranes were exposed to film with a Fusion FX imaging system and processed with a FusionCapt Advance FX7 software program (Beijing Oriental Science and Technology Development Co., Ltd., Beijing, China). Image-Pro Plus 6.0 was utilized to measure the protein concentration (Media Cy-bernetics, Inc., Rockville, MD, USA).

### 5.9. Statistical Analysis

All data were analyzed using the generalized linear model (GLM) method of SAS 9.2 for one-way analysis of variance (SAS Inst. Inc., Cary, NC, USA). Initially, the data were analyzed using a totally random design, with the treatment as the fixed effect and each cell as the random component. For the purpose of determining linear and quadratic responses to the ZEA concentrations, orthogonal polynomial contrasts were utilized. Duncan’s multiple range tests were utilized in order to evaluate the significance of changes between treatments. The threshold for determining significance was set at *p* < 0.05.

## Figures and Tables

**Table 1:** Sequence of primers for real-time PCR.

Target Gene	Primer Sequences (5’ to 3’)	Accession No.
Nrf2	F: GAGTTAGATAGTGCCCCTGGAA R: ACTGGAGCACTATTACCCTGAG	XM_005671981.3
Keap1	F: GTGTGTGCTCCATGTCATGAAT R: CTCCCCAAAGTGCATGTAGATG	NM_001114671.1
Ho1	F: AGGTCCTCAAGAAGATTGCTCA R: CATCTCCAGAGTGTTCATTCGG	NM_001004027.1
Nqo1	F: AAAAGCACTGATCATACTGGCC R: TTCTGGAGATGACGGGATTGAA	NM_ 001159613.1
Sglt1	F: CGTCCATCTTTAACAGCAGCAG R: GCATGTAGATGAAGAGCTGCC	NM_001012297.1
Glut2	F: ACCGACAGCCTATTCTAGTAGC R: AGGAAAACAGAGAGAGCAGTGA	NM_001097417.1
β-actin	F: AGATCACTCCCCCAATGACAG R: AGAGCAAGAGAGGCATCCTG	XM_003124280.5

## Data Availability

Not applicable.
